# Salvianolic acid B ameliorates vascular calcification in rats with chronic kidney disease combined with arteriovenous fistula by inhibiting BMP2/Smads signaling

**DOI:** 10.1080/0886022X.2025.2542524

**Published:** 2025-08-07

**Authors:** Jianhua Ye, Yanmei Li, Xiaolong Yin

**Affiliations:** a Department of Nephrology, General Hospital of Ningxia Medical University, Ningxia, China; b Ningxia Clinical Research Center of Kidney Disease, Ningxia, China

**Keywords:** BMP2/Smads signaling, Salvianolic acid B, vascular calcification, chronic kidney disease, arteriovenous fistula

## Abstract

**Introduction:** Salvianolic acid B (Sal B) can reduce the symptoms of uremia in rats and has a good protective effect on the kidney, but its effect on vascular calcification in chronic kidney disease (CKD) combined with arteriovenous fistula (AVF) is unclear. This study aimed to investigate the effects and mechanisms of Sal B on CKD-AVF.**Methods:** The rat model of CKD-AVF was established and rats were treated with different doses of Sal B. The levels of creatinine (Cr) and urea nitrogen (BUN) in serum were analyzed biochemically; the levels of ALP in serum were measured by ELISA, the pathological damage of kidney and AVF venous segments tissues was observed by HE staining, Masson staining to detect renal fibrosis, and the calcium salt deposition in AVF venous segments tissue was examined by Von Kossa staining, and the protein expression of BMP-2, p-Smad1, Smad1, p-Smad5, Smad5, Osterix, and Runx2 in AVF venous segments tissue was analyzed by Western blot. **Results:** Sal B attenuated the pathological damage of kidney and AVF venous segments tissues, improved calcium salt deposition, reduced the levels of Cr, BUN, and ALP, and inhibited the expression of BMP-2, p-Smad1, p-Smad5, Osterix, and Runx2 in AVF venous segments tissue (*p* < 0.05).**Conclusion:** The mechanism of Sal B inhibition of calcium salt deposition in rats of CKD-AVF may be related to the inhibition of the BMP2/Smads signaling pathway.

## Introduction

Chronic kidney disease (CKD) is a group of diseases characterized by persistently low glomerular filtration rates or markers of renal injury lasting greater than 3 months, the prevalence of which tends to increase from year to year [1]. With the progression of CKD, some patients eventually progress to end-stage renal disease (ESRD) and require renal replacement therapy [2,3]. Maintenance hemodialysis (MHD) is an important means to sustain the life of ESRD patients, and good vascular access is necessary to maintain long-term hemodialysis [4,5]. Arteriovenous fistula (AVF) is the most commonly used permanent vascular access due to its advantages of higher dialysis efficiency, lower infection rate, and longer duration of use, but the high incidence of early dysfunction of AVF poses a serious clinical challenge [6].

AVF dysfunction is mainly characterized by stenosis of venous fistulas due to venous neointimal hyperplasia (VNH), which ultimately reduces dialysis blood flow [7]. Vascular calcification (VC) is closely associated with the development of AVF dysfunction, which can lead to AVF loss of function and increase the risk of inadequate dialysis and death in patients [8,9]. In addition, VC is a prevalent vascular lesion in CKD and chronic inflammatory diseases and is a high-risk factor for mortality [10,11]. There are still no recognized effective treatment options for VC [12]. Therefore, exploring potentially effective preventive and curative measures is of great significance for CKD patients.

In recent years, many Chinese medicinal preparations have been widely used in clinical practice due to their low toxicity and side effects and certain therapeutic effects [13,14]. Salvianolic acid B (Sal B) is the main water-soluble compound in *Salvia miltiorhiza*, which can resist atherosclerosis, anti-inflammation, anti-oxidative stress, improve blood circulation, and reduce tissue ischemia-reperfusion injury [15–17]. The use value of Sal B has gradually received the attention of scholars, and studies have shown that Sal B can improve type 2 diabetes mellitus glucose-lipid metabolism disorders and reduce the damage of diabetic nephropathy on the kidney [18]. Sal B attenuates the symptoms of uremia in rats, and it has a better protective effect on the kidneys [19]. Furthermore, Sal B was able to attenuate diabetic endothelial cell and mitochondrial dysfunction [20]. However, the effect of Sal B on vascular calcification in CKD-AVF is unknown. To investigate the effect of Sal B on vascular calcification in CKD-AVF, the present study intends to establish a rat model of CKD-AVF and intervene with different doses of Sal B to observe its effect and molecular mechanism, to provide a potential option for the treatment of vascular calcification.

## Materials and methods

### Animal experiments

Six-eight weeks old SPF grade SD male rats weighing 180–200 g. Rats were provided by the Experimental Animal Center of Ningxia Medical University, and all animals had free access to water and food. All experimental procedures were approved by the Animal Experimentation Committee and Ethics Committee of General Hospital of Ningxia Medical University (No.2020203), and the number of animals was minimized (*n* = 6). Following completion of the experiments, animals were injected intraperitoneally with sufentanil combined with dexmedetomidine (10 mL/kg, Hengrui Medicine, Jiangsu, China). The animals were executed after inhalation of carbon dioxide to obtain renal tissue, fistula vein segments, and blood.

Model establishment method [21]: After one week of acclimatization feeding in rats, blood was collected from the tail vein to test blood creatinine (Cr) and urea nitrogen (BUN) to determine normal renal function. Subsequently, the rats were randomly divided into control group (Control), model group (CKD-AVF), Sal B low-dose group (Sal B-L), Sal B medium-dose group (Sal B-M), and Sal B high-dose group (Sal B-H). The model rats were first gavaged with 0.6% adenine (200 mg/kg/d, Yuanye Bio, Shanghai) for 4 weeks, followed by 0.3% adenine (200 mg/kg/d) for 2 weeks (induction period), and then 0.15% adenine (200 mg/kg/d) until the end of the experimental period (maintenance period), and then the blood was collected from the tail vein to detect Cr and BUN levels to identify model success.

After the success of the rat CKD model, the left carotid-venous endovascular fistula model (AVF) was established [22]. Briefly, rats were anesthetized by intraperitoneal injection of sufentanil combined with dexmedetomidine (10 mL/kg, Hengrui Medicine, Jiangsu, China), and skin was incised along the midline between the angle of the mandible and the clavicle, with an incision length of about 1.5 cm, and the subcutaneous tissues were separated to reveal the jugular vein. The common carotid artery was isolated between the sternohyoid muscles and the sternothyroideus muscle, and the branches of the artery were ligated. The cervical arteries and veins were brought together in parallel, and the proximal and distal blood flow was blocked with a noninvasive vascular clamp, and the arteries and veins were incised along the long axis of the arteries and veins, and the incision was made at a length of about 0.5 cm, and washed with heparinized saline. After removing the outer membrane, the arteries were anastomosed intermittently, externally, and laterally with 8-0 sutures. After the anastomosis, the distal jugular vein was ligated with a 5-0 suture, and the proximal jugular vein was filled immediately after the opening of the circulation, and obvious pulsations and tremors could be detected. The control group was fed an adenine-free diet and underwent the same procedure as above.

Sal B (Tauto Biotech, Shanghai) was initiated after AVF surgery, and the doses of Sal B-L, Sal B-M, and Sal B-H were 5 mg/kg, 10 mg/kg, and 20 mg/kg, respectively. Sal B was dissolved with 5% dimethyl sulfoxide (DMSO, Yuanye Bio, Shanghai) + 30% PEG300 (Sigma, USA) + 2% Tween80 (Sigma, USA) + H_2_O to dissolve [23,24]. The control group was given an equal amount of saline gavage. After 4 weeks of continuous administration, the rats were executed, and blood, AVF vein segment tissue (within approximately 1 cm of the AVF fistula opening) and kidney tissue were collected for subsequent experiments. In addition, because of the possibility of animal death, we set up our intervention with 10 rats per group, and the final statistical analysis ensured 6 rats per group.

### Biochemical analysis

Serum levels of Cr and BUN were detected by kits (NJJCBio, China). In addition, the levels of alkaline phosphatase (ALP) in serum were determined by ELISA with a double antibody sandwich enzyme-linked immunosorbent method (ZCIBIO, Shanghai). All operations were performed in strict accordance with the manufacturer’s protocol.

### Histopathological observation

The kidney and AVF venous segments were fixed in 4% paraformaldehyde, embedded in paraffin, sectioned (5 µm) and stained with hematoxylin-eosin (HE). The pathological changes of the endothelium and kidney were observed under 200 and 400 times light microscope (Pannoramic 250, Danjier, China), respectively, and scored. The thickness and area of the neointimal were measured by image analysis of the AVF venous segments using Image Pro Plus (IPP) 6.0 software.

### Masson staining

The kidney tissues were fixed with 4% paraformaldehyde, paraffin-embedded, sectioned (5 µm), and Masson stained. Renal fibrosis was observed under a light microscope (Pannoramic 250, Tangier, China), and the blue collagen fiber optical density values in the field of view were analyzed using Image Pro Plus (IPP) 6.0 software.

### Von Kossa staining

The AVF venous segment tissues were fixed with 4% paraformaldehyde, paraffin-embedded, sectioned (5 µm), and Von Kossa (Sunteam Bio, Shanghai) stained. Vascular calcifications were observed under a light microscope (Pannoramic 250, Tangier, China).

### Western blot analysis

Proteins were extracted from AVF venous segments using radioimmuno-precipitation assay buffer (RIPA) (Servicebio, China). Then the protein concentrations were then quantified using the BCA protein assay kit (Beyotime, China) and subjected to SDS-PAGE electrophoresis. And the anti-BMP-2 (1:1000), anti-p-Smad1 (1:2000), anti-Smad1 (1:1000), anti-p-Smad5 (1:2000), anti-Smad5 (1:2000), anti-Osterix (1:2000), and anti-Runx2 (1:2000) antibody (Abclonal, Wuhan) was performed for immune reaction. Finally, a chemiluminescence detection system was used to detect the samples, and ImageJ software (Image-J, National Institutes of Health, USA) was used to quantify the intensity of the bands, β-actin was used as an internal reference.

## Statistical analysis

All data are shown as mean ± standard deviation (SD). The SPSS 19.0 software (SPSS Inc., Chicago, USA) was used to analyze data and statistical differences between groups were determined by one way ANOVA. The LSD-*t* test was used for multiple two-by-two comparisons when the variances were equal, and Tamhane’s T2 test was used when the variances were not equal. *p* < 0.05 were considered statistically significant.

## Results

### Effects of Sal B on renal function and blood pressure in a CKD-AVF model

To verify the effect of Sal B on renal function and blood pressure in the CKD-AVF model, the experiments detected the levels of Cr, BUN, and ALP in the blood of rats. The results, as shown in Table 1, showed that the levels of Cr, BUN, and ALP in the blood of the CKD-AVF group were significantly increased compared with those of the Control group (*p* < 0.01). Compared with the CKD-AVF group, Sal B-M and Sal B-H treatments were able to significantly reduce the levels of Cr, BUN, and ALP in the blood (*p* < 0.01), suggesting that Sal B improves kidney function and blood pressure in the CKD-AVF model.

**Table 1.** The level of Cr, BUN, and ALP in serum (*n* = 6).

Groups	Cr (µmol/L)	BUN (mmol/L)	ALP (gold units/100mL)
Control	47.85 ± 4.58	6.43 ± 0.62	23.78 ± 1.18
CKD-AVF	94.26 ± 6.38**	22.95 ± 2.59**	34.79 ± 0.63**
Sal B-L	79.75 ± 10.71^##^	19.65 ± 3.13	32.42 ± 1.14^#^
Sal B-M	67.80 ± 4.53^##^	16.40 ± 1.72^##^	30.37 ± 0.89^##^
Sal B-H	54.12 ± 2.27^##^	12.71 ± 1.51^##^	26.95 ± 0.86^##^

Data were shown as mean ± SD.

** *p* < 0.01, compared with control group.

^#^ *p* < 0.05.

^##^ *p* < 0.01, compared with CKD-AVF group.

### Effect of Sal B on renal pathology in a CKD-AVF model

**Figure 1.**
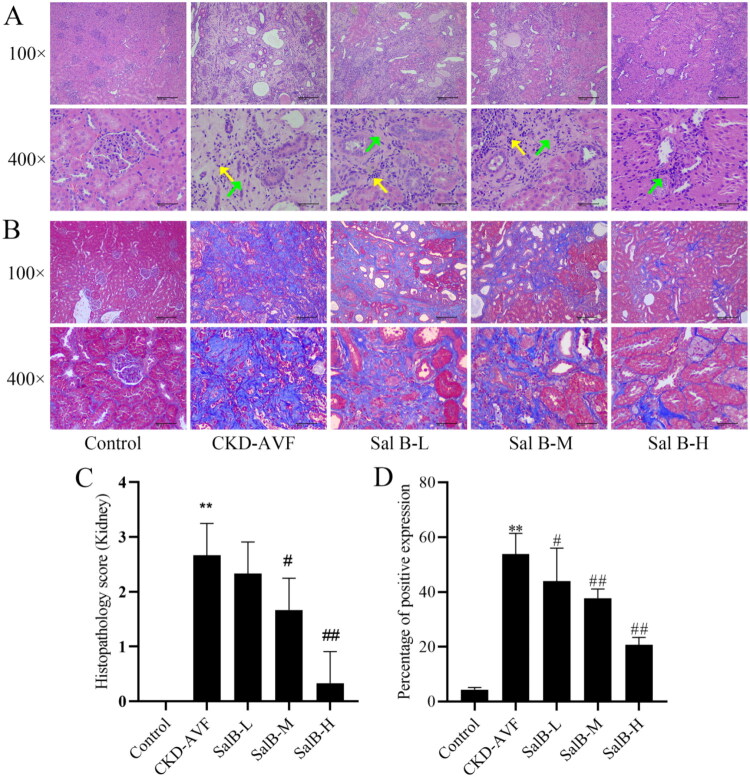
HE staining to observe renal tissue changes in each group, and Masson staining to observe the fibrosis of renal tissue in each group.

To further define the pathological changes of renal tissues in the CKD-AVF model after Sal B intervention, HE staining and Masson staining were performed. The results of HE staining showed that the renal tissues in the Control group showed no obvious pathological changes. The renal tissues of rats in the CKD-AVF group were damaged in structure, with large areas of renal tubules atrophied and necrotic, and the thickening of the basement membrane was obvious. The damage to renal tissues was ameliorated after Sal B treatment at different doses, with a small amount of glomerular involvement, and the histopathologic score was significantly lower after Sal B treatment than in the CKD-AVF group (Figure 1A, C, *p* < 0.05). Masson staining results showed that, compared with the Control group, the collagen fibers (blue) of renal tissues were significantly increased in the CKD-AVF group, and the fibrosis of renal tissues was significantly inhibited in the Sal B-H group compared with the CKD-AVF group (*p* < 0.01, Figure 1B, D), demonstrating that Sal B improved the histopathological injury of renal tissues in the CKD-AVF model.

### Effect of Sal B on neointimal hyperplasia and calcification in a CKD-AVF model

**Figure 2.**
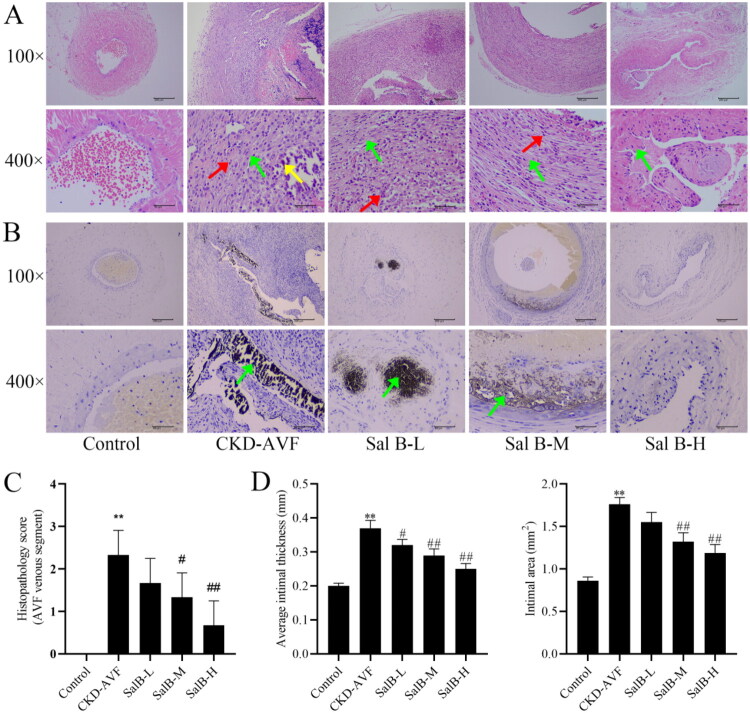
HE staining to observe the neointimal hyperplasia of each group, and Von kossa staining to observe the calcification of each group.

To observe the neointimal hyperplasia and calcification in the CKD-AVF model, HE staining and Von Kossa staining were performed on the tissue of the AVF venous segment. HE staining revealed that the wall thickening of the AVF venous segment was obvious, the lumen was occluded or even disappeared, and a large amount of fibrous tissue and smooth muscle proliferation was observed in the CKD-AVF group when compared with control group. Compared with the CKD-AVF group, the pathologic scores of the AVF venous segments were significantly reduced, wall thickening was significantly attenuated, and endothelial thickness and area were significantly reduced after Sal B treatment (Figure 2A, C, D, *p* < 0.05). Von kossa staining showed significant calcium salt deposition (black or brownish-black) in the CKD-AVF group compared with control group, and Sal B treatment was able to significantly ameliorate calcium salt deposition compared with the CKD-AVF group (Figure 2B), indicating that Sal B reduced neointimal hyperplasia and calcification in the CKD-AVF model.

### Effect of Sal B on the BMP2/smads pathway in vein anastomotic tissue in a CKD-AVF model

**Figure 3.**
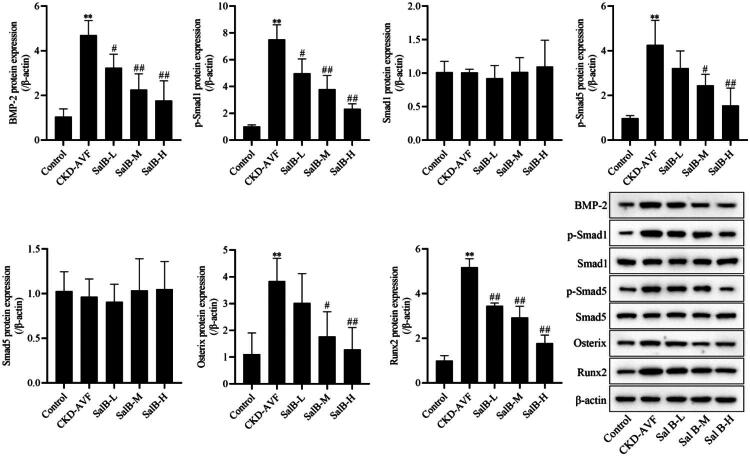
Vascular BMP-2, p-Smad1, p-Smad5, Osterix, Runx2, Smad1, and Smad5 protein expression levels in control and CKD-AVF group. *p < 0.05; **p < 0.01; ***p < 0.001; *p* < 0.0001 compared to the control group.

Changes in BMP2/Smads signaling pathway-related proteins as shown in Figure 3, compared with the control group, the expression of BMP-2, p-Smad1, p-Smad5, Osterix, and Runx2 in the CKD-AVF group increased, and the expression of Smad1 and Smad5 was no significant change. Compared with the CKD-AVF group, Sal B inhibited the expression of BMP-2, p-Smad1, p-Smad5, Osterix, and Runx2 in a dose-dependent manner, indicating that Sal B inhibited the activation of BMP2/Smads signaling pathway in CKD-AVF model.

## Discussion

In the present experiments, we demonstrated that Sal B ameliorated renal injury and alleviated vascular calcification in CKD-AVF rats. Further results showed that Sal B inhibited the activation of the BMP2/Smads signaling pathway. These results suggest that Sal B may achieve amelioration of injury in CKD-AVF rats by inhibiting BMP2/Smads signaling pathway.

An animal model that can fully simulate the clinic is the key to research, and the CKD-AVF model was established in this experiment. The CKD model is usually established by surgery, but the surgical method is more complicated, and the surgery is easy to cause bleeding, infection, and even model death, in addition, the model has a large individual difference, which affects the later experimental research [25,26]. Compared with surgical intervention, adenine induction is a noninvasive chemical method, and its gradual feeding makes it easy for animals to tolerate and has a high survival rate [27]. It was concluded that the abnormal metabolism produced in rats fed adenine for a long period is similar to the clinical manifestations and pathologic changes of chronic renal failure in humans [28]. In this study, we first used adenine induction to establish a CKD model, and based on this, we established an AVF model. It was observed that CKD-AVF rats showed a significant increase in Cr, BUN, and ALP levels, obvious damage to renal tissue structure, large atrophy and necrosis of renal tubules, obvious thickening of the tubular wall in the venous segment of AVF, occlusion or even disappearance of the tubular lumen, which was similar to the human clinicopathological changes [29], indicating that the simulated CKD-AVF model was successful.

Studies have reported that MHD patients are more likely to develop arterial calcification than the general population, with 40% of uremic patients having arterial microcalcifications prior to AVF [30]. It is currently believed that AVF calcification is positively correlated with dialysis age [31]. Arterial calcification limits the expansion of the arterial lumen after AVF surgery, thus prolonging the maturation time of AVF [32]. Meanwhile, vascular calcification can significantly increase the probability of AVF dysfunction in MHD patients and is also an independent risk factor for AVF dysfunction [33]. Compared with non-calcified vessels, vessels with severe calcification are less elastic and more brittle, and are more prone to complications such as intra-organic thrombus and anastomotic non-healing after AVF surgery [34]. Therefore, AVF calcification directly affects AVF maturation and patient survival. In this experiment, CKD-AVF rats showed significant calcium salt deposition in AVF venous segment, and Sal B treatment significantly ameliorated calcium salt deposition; moreover, AVF venous segment and renal histopathological damage were significantly improved by Sal B treatment, which suggests that Sal B attenuates calcification and ameliorates tissue damage in CKD-AVF rats.

There is a close relationship between the BMP2/Smads/Runx2/Osterix signaling pathway, a co-regulator of vascularity and bone formation, and vascular calcification [35]. BMP2 is a member of the transforming growth factor β superfamily, and its receptor is a heterodimer consisting of two subunits, type I and type II. The Smad protein family is divided into receptor-activated Smad (R-Smad, e.g., Smad1 and Smad5), co-mediated Smad (Co-Smad, e.g., Smad4), and inhibitory Smad (I-Smad, e.g., Smad6 and Smad7). Upon binding to its receptor, BMP2 phosphorylates R-Smad (Smad1 and Smad5), and the phosphorylated R-Smad is subsequently translocated to the nucleus to regulate the transcription of target genes. In addition, Smads promote phosphorylation of Runx2 proteins through their distal P1 promoter and proximal P2 promoter [36,37]. Osterix is a downstream molecule of Runx2, and Runx2 enhances Osterix expression [38], but in Runx2 knockout mice, activation of BMP2 also induces the expression of Osterix and ALP, and up-regulation of Smad1 expression also promotes Osterix expression [39]. Since the formation process of vascular calcification is similar to bone and cartilage development [40], it has also been shown that BMP2 signaling is involved in the progression of vascular calcification by playing an important role in vascular calcification [41,42]. In the present study, the expression of BMP-2, p-Smad1, p-Smad5, Osterix, and Runx2 was significantly increased in CKD-AVF rats, suggesting that BMP2/Smads/Runx2/Osterix signaling may be involved in the progression of CKD-AVF. Sal B inhibited the expression of BMP-2, p-Smad1, p-Smad5, Osterix, and Runx2 in a dose-dependent manner, demonstrating that Sal B may exert a protective effect on CKD-AVF rats by inhibiting BMP2/Smads/Runx2/Osterix signaling.

## Conclusion

In conclusion, the results of this study demonstrated that Sal B ameliorated renal injury and alleviated vascular calcification in CKD-AVF rats, inhibited the activation of the BMP2/Smads signaling pathway, Sal B may ameliorate injury in CKD-AVF rats by inhibiting the BMP2/Smads signaling pathway. However, a limitation of the study is the uncertainty regarding whether Sal B indeed mitigates damage in CKD-AVF rats *via* the inhibition of BMP2/Smads signaling. To strengthen the conclusions drawn from this research, we will perform validation experiments using BMP2/Smads signaling inhibitors in future research work to verify the proposed mechanism of action.

## Data Availability

The datasets used and/or analyzed during the current study are available from the corresponding author upon reasonable request.
